# Supplementary material for: Reaching Natural Growth: The Significance of Light and Temperature Fluctuations in Plant Performance in Indoor Growth Facilities
Source: Plants (Basel). 2020 Oct 5;9(10):1312. doi: 10.3390/plants9101312 (PMC7600060; doi:10.3390/plants9101312)
Supplement: Supplementary file 1 [file plants-09-01312-s001.pdf]

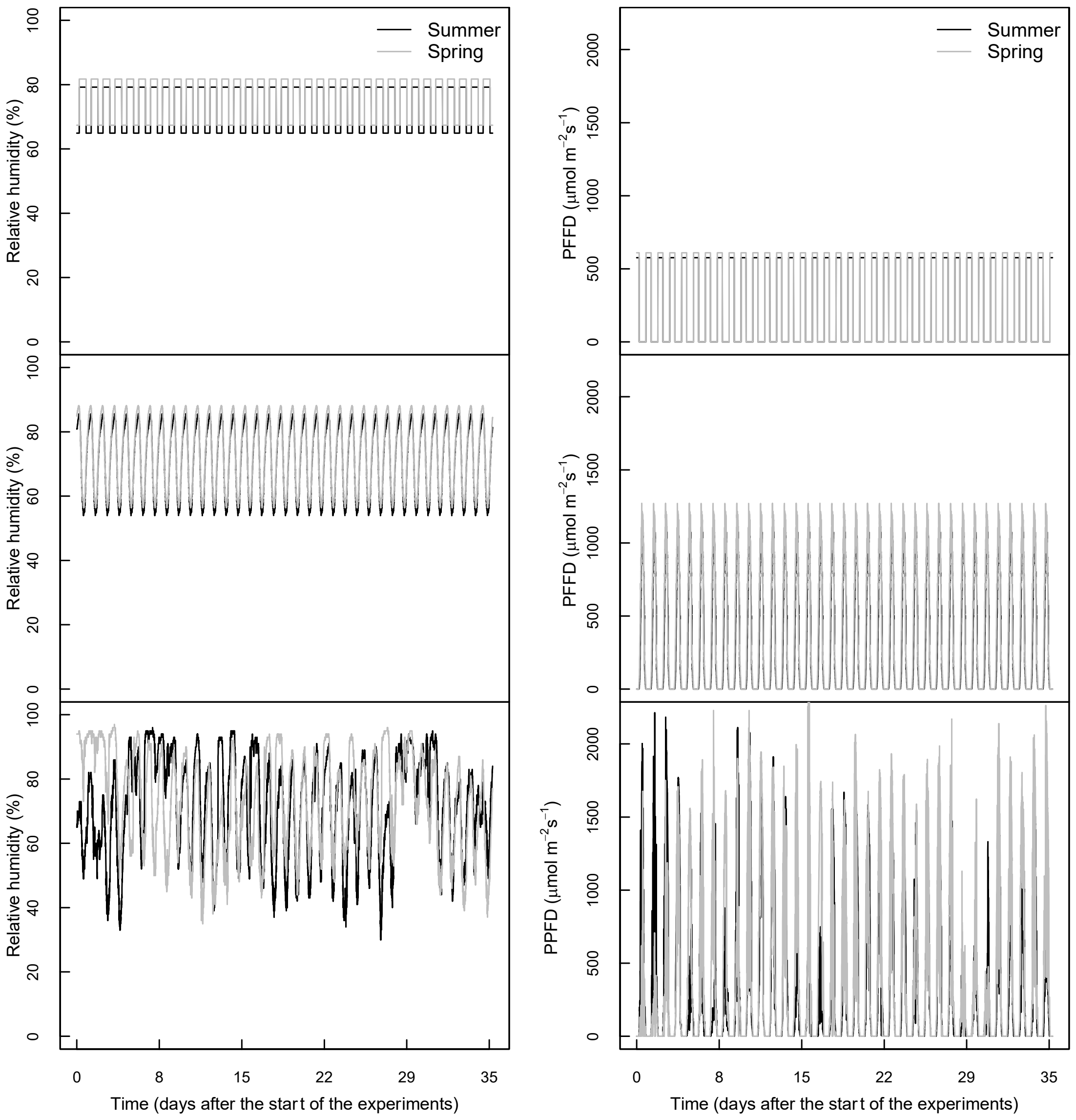

Figure 1. Applied relative humidity (%) and PPFD (μmol m^{-2} s^{-1}) for each treatment in summer or spring conditions

.

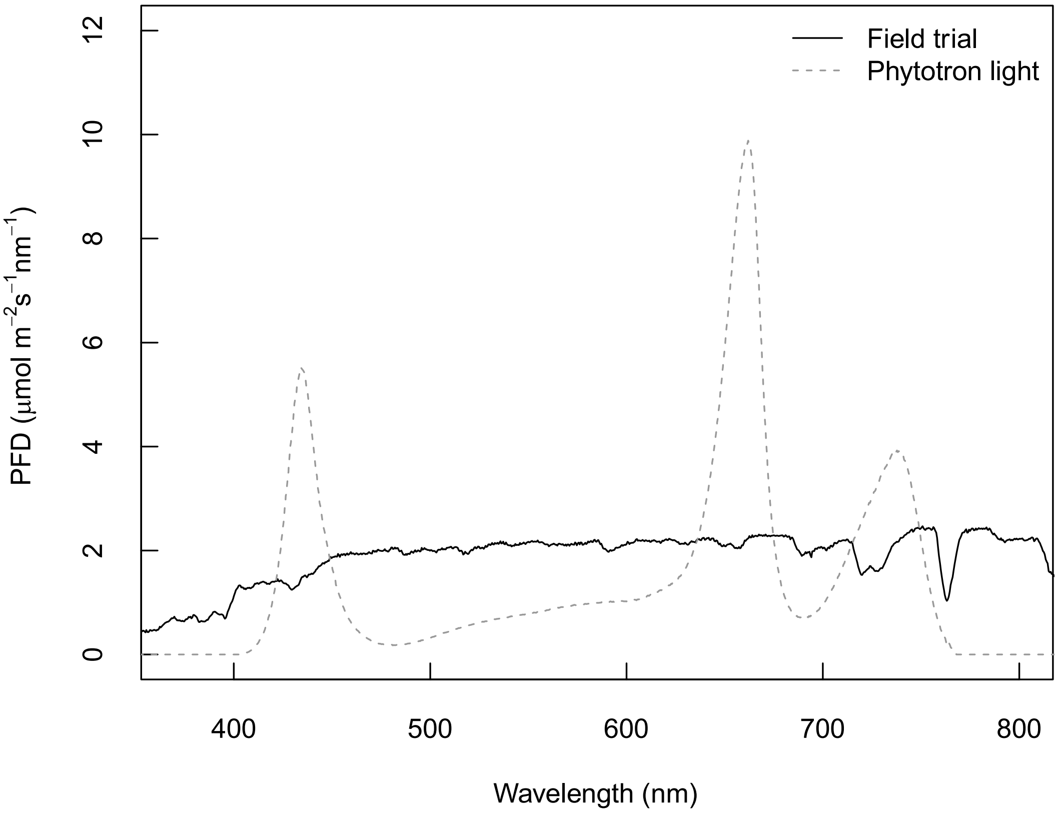

Figure 2. Spectrum examples of the applied light. The field trial example corresponds to a sample of the sun spectra (28% Blue light, 36% Green, 36% Red and R:FR 1.1 in average), meanwhile the phytotron light quality corresponds to the used spectra in the phytotrons (25%B, 16%G, 59%R and R:FR 1.8). The integrated area between 400 and 700 nm corresponds to an approximately 575 μmol $m^{-2}s^{-1}$ of photosynthetic photon flux density in each case

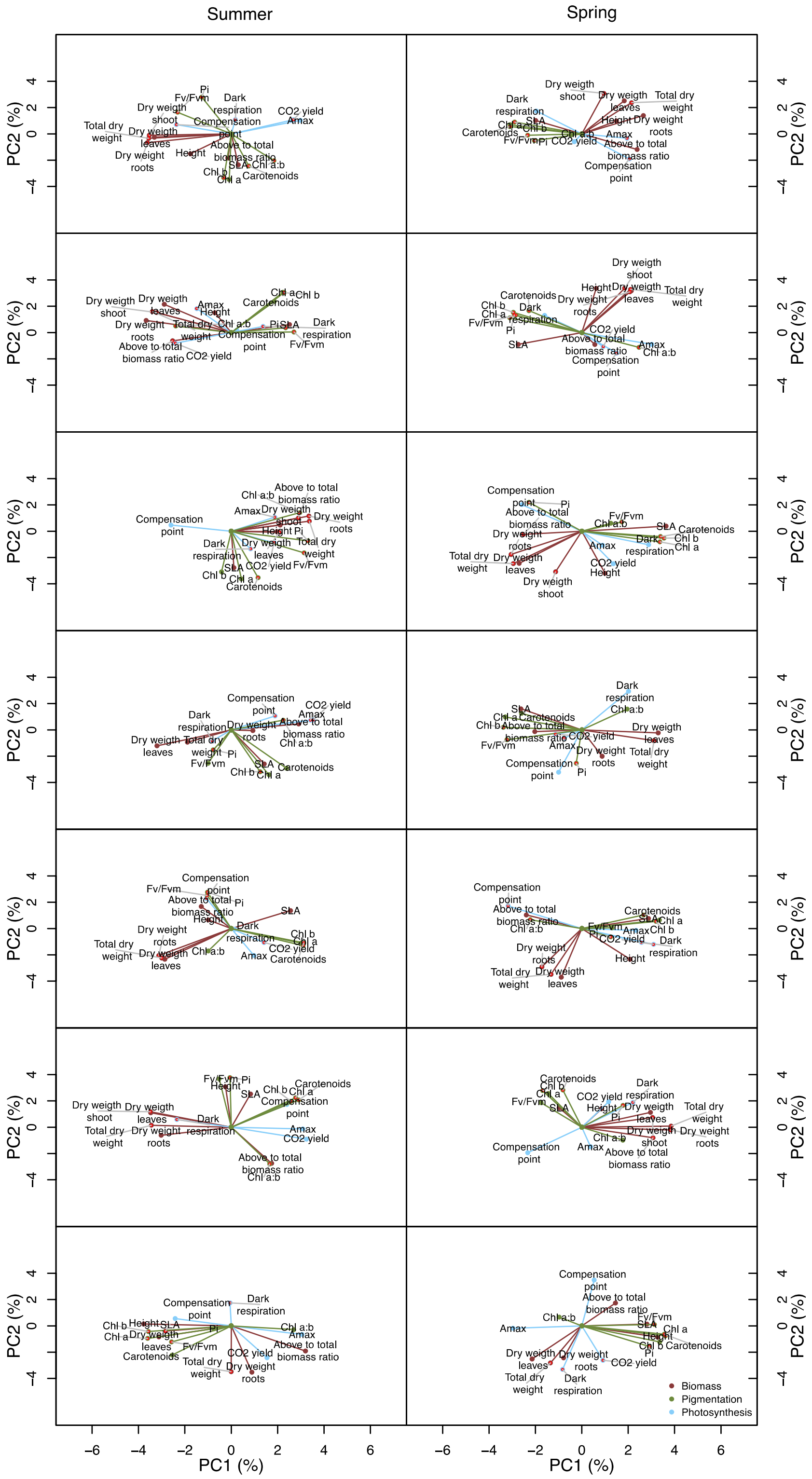

Figure 3. Principal component analysis loadings of each species in two different conditions: Summer and Spring. *Alnus* was not included in the spring trial under the sinusoidal treatment

Species	Alnus				Ulmus			
**Trial¥Treatment**	**Outdoor**	**Fix**	**Sinusoidal**	**Variable**	**Outdoor**	**Fix**	**Sinusoidal**	**Variable**
Biomass and Morphology								
Height*	10.88±1.3	13.34±2.09	-	9.07±1.2	26.19±4.48	33.41±3.92	40.18±3.8	36.44±3.82
Dry weight leaves	0.25±0.04	0.27±0.03	-	0.2±0.05	1.42±0.32	3.1±0.91	1.96±0.38	1.98±0.35
Dry weight shoot	0.08±0.01	0.13±0.01	-	0.1±0.03	0.78±0.23	1.31±0.4	1.18±0.28	0.96±0.19
Dry weight roots	0.23±0.04	0.25±0.02	-	0.11±0.03	1.29±0.22	1.52±0.43	1.37±0.33	1.13±0.19
Total dry weight	0.55±0.09	0.64±0.06	-	0.42±0.1	3.49±0.74	5.93±1.73	4.52±0.97	4.07±0.72
Root to Shoot ratio	0.72±0.07	0.62±0.02	-	0.35±0.05	0.61±0.06	0.36±0.05	0.42±0.03	0.37±0.03
SLA	31.76±0.04	21.04±0.65	-	49.35±9.37	25.31±1.65	22.35±1.44	27.35±0.93	29.99±2.69
Chlorophyll								
Chlorophyll a (mg g-1)	2.72±0.16	2.5±0.28	-	6.89±0.47	2.55±0.41	1.65±0.26	3.07±0.28	4.29±0.62
Chlorophyll b (mg g-1)	0.64±0.03	0.64±0.1	-	1.7±0.18	0.61±0.11	0.38±0.05	0.73±0.07	1.13±0.18
Chl a: b ratio	1.34±0.14	1.45±0.14	-	2.59±0.12	0.97±0.13	0.79±0.09	1.1±0.13	1.24±0.17
Carotenoids (mg g-1)	4.25±0.06	4.01±0.25	-	4.1±0.25	4.23±0.09	4.29±0.1	4.2±0.1	3.8±0.1
Fv/Fm	0.79±0.01	0.7±0.02	-	0.82±0.01	0.8±0	0.75±0.01	0.81±0.01	0.82±0.01
Photosynthesis								
Max photosynthesis**	9.73±1.36	10.43±2.62	-	8.23±1.6	6.99±0.6	9.95±0.68	7.26±0.42	5.86±0.41
Initial slope	0.052±0.006	0.041±0.007	-	0.048±0.004	0.04±0.004	0.044±0.001	0.039±0.002	0.04±0.002
Dark respiration	-4.08±0.18	-2.04±0.16	-	-1.24±0.22	-3.1±0.55	-1.92±0.17	-2.21±0.13	-1.39±0.07
Compensation point	79.67±6.77	50±11.59	-	22±4.04	74±4.73	42±4.36	53±1.73	29.33±1.33

Table S1: Absolute values of all measured traits for each species and treatment in the 'summer' run. Values are means ± s.e., *n* = 3 to 9 (see methods for details).

Species	Ocimum				Lactuca			
Trial¥Treatment	Outdoor	Fix	Sinusoidal	Variable	Outdoor	Fix	Sinusoidal	Variable
Biomass and Morphology								
Height*	17.28±0.33	22.14±1.45	20.09±1.51	25.91±1.82	-	-	-	-
Dry weight leaves	1.48±0.15	2.28±0.3	1.3±0.13	1.06±0.17	6.99±1.07	22.04±3.2	6.93±0.46	6.22±0.9
Dry weight shoot	0.38±0.05	0.55±0.08	0.34±0.04	0.48±0.1	-	-	-	-
Dry weight roots	1.2±0.22	1.68±0.3	0.67±0.21	0.45±0.12	5.79±1.32	5.33±1.04	5.6±0.71	2.6±0.59
Total dry weight	3.05±0.38	4.52±0.63	2.31±0.28	1.99±0.35	12.78±2.27	27.36±3.68	12.52±1.08	8.83±1.11
Root to Shoot ratio	0.63±0.09	0.59±0.07	0.42±0.13	0.26±0.05	0.79±0.1	0.29±0.06	0.8±0.07	0.71±0.32
SLA	19.67±1.16	14.03±1.27	25.47±0.7	32.71±1.79	36.84±2.39	31±3.12	69.54±9.03	65.77±7.05
Chlorophyll								
Chlorophyll a (mg g-1)	2.52±0.15	1.6±0.29	3.41±0.13	5.45±0.52	3.52±0.37	1.9±0.22	5.78±0.56	4.76±0.48
Chlorophyll b (mg g-1)	0.54±0.04	0.36±0.07	0.75±0.06	1.14±0.11	0.66±0.09	0.34±0.03	1.1±0.11	0.93±0.14
Chl a: b ratio	0.86±0.04	0.52±0.08	0.99±0.03	1.41±0.1	1.23±0.09	0.92±0.1	1.97±0.23	1.58±0.17
Carotenoids (mg g-1)	4.75±0.14	4.57±0.11	4.61±0.25	4.78±0.11	5.45±0.23	5.57±0.28	5.29±0.32	5.2±0.46
Fv/Fm	0.83±0.01	0.83±0	0.84±0.01	0.84±0	0.85±0	0.83±0.01	0.86±0	0.85±0
Photosynthesis								
Max photosynthesis**	9.23±1.31	12.64±1.01	13.5±1.03	12.22±1.96	5.17±0.18	7.66±0.69	10.39±1.82	7.56±1.91
Initial slope	0.039±0.004	0.054±0.002	0.051±0.002	0.055±0.004	0.04±0.007	0.041±0.004	0.051±0.004	0.046±0.006
Dark respiration	-2.89±0.28	-2.8±0.28	-2.7±0.56	-1.43±0.18	-3.53±0.61	-1.53±0.07	-2.88±0.32	-1.49±0.6
Compensation point	70.67±5.9	49±7.51	52±11.85	24.67±2.73	77.33±1.86	35.67±4.06	55.33±0.88	23.67±9.21

Table S1 (continuation): Absolute values of all measured traits for each species and treatment in the 'summer' run. Values are means ± s.e., *n* = 3 to 9 (see methods for details).

.

Species	Melissa				Raphanus			
**Trial¥Treatment**	**Outdoor**	**Fix**	**Sinusoidal**	**Variable**	**Outdoor**	**Fix**	**Sinusoidal**	**Variable**
Biomass and Morphology								
Height*	14.4±1.83	22.22±0.95	20.37±1.81	27.08±1.59	7.57±1.29	8.6±0.97	6.77±0.58	9.5±0.85
Dry weight leaves	0.78±0.18	1.56±0.19	1.39±0.37	1.01±0.12	2.83±0.23	4.66±0.16	2.74±0.22	4.06±0.28
Dry weight shoot	-	-	-	-	0.58±0.1	1.28±0.13	0.51±0.05	0.88±0.1
Dry weight roots	0.87±0.23	0.78±0.09	1.16±0.32	0.5±0.06	4.51±0.35	15.94±0.44	6.04±0.64	6.13±0.54
Total dry weight	1.65±0.41	2.34±0.28	2.55±0.68	1.5±0.18	7.92±0.58	21.88±0.56	9.29±0.86	11.07±0.78
Root to Shoot ratio	1.04±0.08	0.5±0.02	0.82±0.05	0.5±0.01	1.35±0.08	2.72±0.14	1.84±0.13	1.26±0.09
SLA	32.02±2.15	26.72±2.24	37.44±1.98	46.08±1.44	25.87±2.93	25.73±1.55	32.05±2.07	35.17±3.81
Chlorophyll								
Chlorophyll a (mg g-1)	4.01±0.41	2.88±0.27	4.06±0.25	7.34±0.95	3.71±0.45	2.73±0.33	3.21±0.24	5.68±0.73
Chlorophyll b (mg g-1)	0.9±0.1	0.65±0.05	0.96±0.05	1.81±0.26	0.91±0.08	0.65±0.07	0.94±0.12	1.43±0.29
Chl a: b ratio	1.55±0.16	1.11±0.09	1.45±0.08	2.48±0.44	1.07±0.08	1.03±0.15	1.22±0.16	1.67±0.26
Carotenoids (mg g-1)	4.47±0.12	4.38±0.07	4.24±0.07	4.1±0.27	4.05±0.16	4.19±0.22	3.52±0.39	4.1±0.37
Fv/Fm	0.82±0	0.81±0	0.83±0.01	0.83±0	0.83±0	0.82±0.01	0.85±0	0.84±0.01
Photosynthesis								
Max photosynthesis**	10.61±0.84	12.08±1.39	12.46±0.56	13.35±0.86	11.86±1.55	10.84±1.24	7.87±2.1	10.33±0.57
Initial slope	0.041±0.003	0.049±0.002	0.052±0.004	0.062±0.001	0.049±0.005	0.06±0.002	0.052±0.005	0.06±0.002
Dark respiration	-2.76±0.19	-1.98±0.25	-2.13±0.08	-1.57±0.1	-3±0.24	-1.54±0.16	-1.75±0.28	-1.81±0.23
Compensation point	66.67±2.96	39.33±6.49	38.67±5.17	23.33±2.4	56.67±6.84	22.67±3.28	29±3.51	27±3.21

Table S1. (continuation): Absolute values of all measured traits for each species and treatment in the 'summer' run. Values are means ± s.e., n = 3 to 9 (see methods for details).

.

Species	Triticum			
**Trial¥Treatment**	**Outdoor**	**Fix**	**Sinusoidal**	**Variable**
Biomass and Morphology				
Height*	54.06±1.59	45.74±1.71	57.82±1.06	62.64±1.38
Dry weight leaves	10.35±1.2	29.54±1.89	20.67±1.43	21.67±3.75
Dry weight shoot	-	-	-	-
Dry weight roots	63.53±5.66	83.84±8.76	68.29±4.9	88.07±9.38
Total dry weight	73.87±6.35	113.38±9.66	88.96±5.57	109.73±10.77
Root to Shoot ratio	6.78±0.89	2.89±0.28	3.39±0.3	4.5±0.6
SLA	34.59±3.89	18.48±1.38	26.28±1.4	35.66±1.74
Chlorophyll				
Chlorophyll a (mg g-1)	5.8±0.29	1.96±0.25	4.68±0.41	8.25±1.06
Chlorophyll b (mg g-1)	1.39±0.11	0.47±0.06	1.17±0.07	2.13±0.35
Chl a: b ratio	1.77±0.08	1.04±0.06	1.54±0.13	2.48±0.25
Carotenoids (mg g-1)	4.19±0.13	4.17±0.09	3.97±0.17	3.95±0.17
Fv/Fm	0.82±0.01	0.8±0.01	0.84±0.01	0.83±0
Photosynthesis				
Max photosynthesis**	9.21±1.62	13.86±0.57	9.03±1.02	8.58±2.83
Initial slope	0.049±0.001	0.058±0.003	0.056±0.004	0.065±0.009
Dark respiration	-4.46±0.25	-1.41±0.11	-2.78±0.57	-1.18±0.15
Compensation point	88.33±9.74	23.33±1.33	44.67±5.67	12.67±1.45

Table S1 (continuation): Absolute values of all measured traits for each species and treatment in the 'summer' run. Values are means ± s.e., n = 3 to 9 (see methods for details).

Species	Alnus				Ulmus			
**Trial¥Treatment**	**Outdoor**	**Fix**	**Sinusoidal**	**Variable**	**Outdoor**	**Fix**	**Sinusoidal**	**Variable**
Biomass and Morphology								
Height*	22.32±1.2	9.88±1.57	15.96±1.91	13.37±2.17	26.67±3.05	24.46±1.64	24.62±4.8	25.59±3.27
Dry weight leaves	1.06±0.1	0.37±0.07	0.93±0.24	0.32±0.07	1.44±0.28	2.02±0.16	0.93±0.17	0.68±0.16
Dry weight shoot	0.51±0.05	0.17±0.04	0.6±0.16	0.16±0.04	0.71±0.15	1.26±0.15	0.63±0.13	0.38±0.09
Dry weight roots	0.52±0.05	0.11±0.04	0.6±0.16	0.24±0.05	0.63±0.14	1.91±0.21	0.65±0.15	0.59±0.18
Total dry weight	2.09±0.19	0.65±0.09	2.13±0.55	0.72±0.16	2.77±0.56	5.19±0.46	2.21±0.43	1.65±0.42
Root to Shoot ratio	0.34±0.03	0.25±0.08	0.39±0.02	0.57±0.07	0.28±0.02	0.59±0.05	0.4±0.03	0.5±0.08
SLA	34.76±1.24	21.83±4.19	26.83±3.18	38.07±2.08	28.71±0.93	22.1±0.43	25.72±2.16	31.16±1.05
Chlorophyll								
Chlorophyll a (mg g-1)	7.35±0.97	3.02±0.13	1.69±0.41	5.76±0.43	6.8±0.43	1.8±0.07	1.47±0.39	4.28±0.18

Chlorophyll b (mg g-1)	1.45±0.25	0.64±0.04	0.43±0.13	1.24±0.13	1.54±0.09	0.36±0.02	0.34±0.08	1±0.03
Chl a: b ratio	1.94±0.18	1.89±0.1	0.94±0.39	2.09±0.15	1.15±0.09	0.64±0.04	0.53±0.13	1.26±0.07
Carotenoids (mg g-1)	5.12±0.23	4.72±0.11	4.08±0.2	4.68±0.15	4.42±0.05	5±0.16	4.26±0.17	4.3±0.08
Fv/Fm	0.78±0.01	0.77±0.03	0.81±0	0.77±0	0.81±0	0.77±0.01	0.81±0.01	0.79±0.01
Photosynthesis								
Max photosynthesis**	8.37±0.29	14.25±0.68	5.81±1.58	9.17±0.52	7.3±0.39	9.62±1.3	5.04±1.65	9.36±1.2
Initial slope	0.044±0.002	0.059±0.002	0.04±0.004	0.045±0.003	0.034±0.002	0.052±0.002	0.038±0.004	0.046±0.003
Dark respiration	-1.74±0.07	-1.77±0.08	-1.8±0.59	-1.72±0.14	-1.27±0.15	-1.59±0.14	-1.52±0.27	-1.48±0.14
Compensation point	30.33±2.67	27.33±1.76	41.67±15.68	35±2.52	30.33±0.88	27±4.58	31.67±6.01	27.67±2.85

Table S2: Absolute values of all measured traits for each species and treatment in the 'spring' run**.** Values are means ± s.e., *n* = 3 to 9 (see methods for details).

**Species**		**Ocimum**				**Lactuca**		
**Trial¥Treatment**	**Outdoor**	**Fix**	**Sinusoidal**	**Variable**	**Outdoor**	**Fix**	**Sinusoidal**	**Variable**
Biomass and Morphology								
Height*	21.16±1.38	24.86±1.25	25.62±1.2	18.48±0.45	-	-	-	-
Dry weight leaves	1.25±0.14	1.77±0.15	1.63±0.19	1.27±0.14	8.79±0.54	6.62±0.46	13.08±0.75	4.89±0.29
Dry weight shoot	0.38±0.04	0.54±0.04	0.54±0.06	0.27±0.03	-	-	-	-
Dry weight roots	0.37±0.05	1.62±0.15	1.12±0.26	0.91±0.21	3.15±0.31	6.86±0.56	5.98±0.9	5.28±1.04
Total dry weight	2±0.22	3.92±0.2	3.29±0.35	2.45±0.33	11.94±0.82	13.48±0.78	19.06±1.48	10.17±1.15
Root to Shoot ratio	0.23±0.02	0.73±0.08	0.53±0.11	0.58±0.11	0.35±0.02	1.06±0.1	0.45±0.06	1.08±0.19
SLA	21.37±1.19	13.62±0.54	43.17±2	18.09±0.64	25.14±1.77	27.19±1.29	38.21±2.58	43.91±3.09
Chlorophyll								
Chlorophyll a (mg g-1)	2.19±0.29	1.97±0.24	4.07±0.42	3.92±0.39	2.51±0.53	1.59±0.14	3.29±0.05	4.64±0.37
Chlorophyll b (mg g-1)	0.54±0.08	0.37±0.05	0.84±0.11	0.84±0.12	0.57±0.12	0.28±0.03	0.68±0.03	0.91±0.07
Chl a: b ratio	0.55±0.08	0.64±0.07	1.25±0.15	1.18±0.08	0.68±0.14	0.72±0.06	1.08±0.05	1.57±0.12
Carotenoids (mg g-1)	4.08±0.05	5.38±0.26	4.9±0.15	4.75±0.16	4.38±0.33	5.76±0.32	4.92±0.2	5.11±0.13
Fv/Fm	0.77±0.02	0.82±0.01	0.85±0	0.82±0.01	0.85±0.01	0.84±0.01	0.86±0	0.85±0.01
Photosynthesis								
Max photosynthesis**	6.73±0.3	16.46±1.06	8.45±2.4	19.19±0.42	5.77±0.65	13.5±2.71	3.64±1.19	16±1.38
Initial slope	0.037±0.001	0.057±0.005	0.046±0.006	0.057±0.001	0.038±0.002	0.051±0.003	0.032±0.009	0.061±0.001
Dark respiration	-2.8±0.21	-2.18±0.07	-2.17±0.49	-1.97±0.02	-1.85±0.17	-1.88±0.35	-1.65±0.61	-2.15±0.35
Compensation point	76.33±3.38	37.33±3.33	47.67±18.19	34.33±0.33	38.33±6.12	35.67±7.31	36±7.02	34±5.77

Table S2 (continuation): Absolute values of all measured traits for each species and treatment in the 'spring' run. Values are means ± s.e., *n* = 3 to 9 (see methods for details).

**Species**		**Melissa**				**Raphanus**		
**Trial¥Treatment**	**Outdoor**	**Fix**	**Sinusoidal**	**Variable**	**Outdoor**	**Fix**	**Sinusoidal**	**Variable**

Biomass and Morphology								
Height*	25.91±0.81	17.27±0.64	23.21±1.91	16.8±1.5	5.94±0.2	4.36±0.14	6.64±0.27	6.97±0.46
Dry weight leaves	3.62±0.66	4.28±0.3	1.73±0.46	0.87±0.14	2.25±0.18	1.27±0.14	3.42±0.25	1.12±0.06
Dry weight shoot	-	-	-	-	0.53±0.05	0.22±0.02	0.67±0.03	0.24±0.03
Dry weight roots	2.1±0.39	3.01±0.21	1.24±0.33	0.55±0.1	4.7±0.53	4.54±0.44	5.94±0.31	2.66±0.25
Total dry weight	5.72±1.04	7.29±0.48	2.97±0.78	1.42±0.22	7.47±0.68	6.04±0.58	10.03±0.57	4.02±0.32
Root to Shoot ratio	0.58±0.02	0.71±0.04	0.74±0.04	0.63±0.07	1.69±0.13	3.07±0.16	1.47±0.05	1.95±0.13
SLA	40.16±2.58	20.59±0.67	46.87±3.39	53.3±9.11	23±0.92	24.95±0.41	30.99±1.74	31.21±2.05
Chlorophyll								
Chlorophyll a (mg g-1)	8.04±1.54	3.23±0.22	4.49±0.68	8.2±0.88	2.72±0.8	3.3±0.26	2.83±0.35	7.81±0.61
Chlorophyll b (mg g-1)	1.82±0.3	0.72±0.06	1.09±0.16	2.02±0.24	0.67±0.16	0.78±0.07	0.82±0.1	2.09±0.31
Chl a: b ratio	1.94±0.39	1.08±0.08	1.53±0.25	2.56±0.23	0.63±0.18	1.04±0.02	0.97±0.1	2.22±0.19
Carotenoids (mg g-1)	4.35±0.18	4.49±0.15	4.11±0.16	4.08±0.07	3.92±0.24	4.26±0.11	3.48±0.02	3.84±0.24
Fv/Fm	0.81±0.01	0.81±0	0.83±0.01	0.8±0.01	0.83±0.01	0.8±0	0.85±0	0.85±0.01
Photosynthesis								
Max photosynthesis**	11.08±0.33	13.45±1.12	9.1±2.3	14.93±0.38	13.77±1.97	15.53±1.61	7.17±1.73	19.7±2.01
Initial slope	0.051±0.004	0.057±0.003	0.051±0.007	0.063±0.001	0.046±0.003	0.069±0.001	0.054±0.009	0.069±0.002
Dark respiration	-1.63±0.09	-1.67±0.15	-1.68±0.3	-1.51±0.05	-1.05±0.01	-1.76±0.23	-1.35±0.15	-1.95±0.28
Compensation point	24.33±1.2	26.33±3.53	32±9.71	22.33±0.88	22.33±1.33	24±3.06	20.33±0.67	27.67±3.84

Table S2 (continuation): Absolute values of all measured traits for each species and treatment in the 'spring' run**.** Values are means ± s.e., *n* = 3 to 9 (see methods for details).

**Species**	**Triticum**			
**Trial¥Treatment**	**Outdoor**	**Fix**	**Sinusoidal**	**Variable**
Biomass and Morphology				
Height*	53.96±1.21	28.68±0.57	54.08±1.21	43.71±1.04
Dry weight leaves	12.29±0.97	6.73±0.44	18.85±0.64	8.11±0.39
Dry weight shoot	-	-	-	-
Dry weight roots	21.6±2.49	47.88±4.56	54.92±3.56	48.41±5.81
Total dry weight	33.89±2.52	54.6±4.77	73.77±4.02	56.52±5.96
Root to Shoot ratio	1.86±0.29	7.24±0.74	2.91±0.15	5.99±0.62
SLA	30.38±0.63	16.91±0.62	33.34±4.18	27.83±1.61
Chlorophyll				
Chlorophyll a (mg g-1)	7.12±0.44	3.04±0.26	7.29±0.47	6.51±0.51
Chlorophyll b (mg g-1)	1.89±0.07	0.67±0.06	1.8±0.12	1.64±0.13
Chl a: b ratio	1.45±0.05	1.23±0.14	2.15±0.22	1.93±0.13
Carotenoids (mg g-1)	3.75±0.12	4.53±0.06	4.04±0.04	3.97±0.04
Fv/Fm	0.83±0	0.81±0.02	0.84±0	0.83±0
Photosynthesis				

Max photosynthesis**	9.12±0.47	18.19±5.14	11.26±3.14	14.02±1.48
Initial slope	0.044±0	0.081±0.013	0.068±0.004	0.098±0.012
Dark respiration	-1.9±0.05	-2.12±0.5	-2.72±0.41	-3.44±1.24
Compensation point	35±0	23±3.51	37.67±6.17	26.33±4.63

Table S2 (continuation): Absolute values of all measured traits for each species and treatment in the 'spring' run. Values are means ± s.e.,n = 3 to 9 (see methods for details)

plants

MDPI
